# True Ringworm

**Published:** 1861-05

**Authors:** Jona. Hutchison

**Affiliations:** The London and Metropolitan Hospitals.—Extract


### TRUE RINGWORM.

BY JONA. HUTCHISON,

*Of the London and Metropolitan Hospitals.—Extract.*

*Treatment.*—From time immemorial it has been customary to employ various local irritants for the cure of ringworm. Ink is the favorite application of mothers, and is a scientific and frequently successful remedy. At the Hospital for Skin diseases, Mr. Startin always blisters the patches with the vesicating fluid. A single blistering is usually sufficient for patches on the skin, but those on the scalp often require two or more. Many surgeons employ nitrate of silver in solid stick. M. Bazin insists strongly on epilation as a means of cure, and there can be no doubt but that it is an extremely important one. By removal of the affected and adjacent hairs we can reduce a ringworm patch on the scalp to the same condition as one of the general surface, thus rendering it much more accessible to the influence of parasiticides. Of the latter it probably does not matter much which we select. The creosote ointment is a very good one, so also is the application of strong acetic acid. The great point in treatment is to keep clearly in mind that the destruction of a vegetable parasite is the object aimed at. It is needless to point out how strongly the fact that ringworm is curable by local means supports the opinions held in this report as its purely local pathology.

*Conclusions.*—1. True ringworm, or tinea tonsurans, may be defined as a disease affecting either the scalp or the general surface, in which circular patches are formed, on which the hairs break off short, and a short, and a slight, branny desquamation is seen, both hairs and epidermic scales exhibiting under the microscope the sporules and thalli of a fungus.

2. Ringworm in the scalp is rarely seen, excepting in children; but on the general surface is not very unfrequent in young adults.

3. It is contagious, and spreads by contagion only.

4. It is not attended by any peculiar form of dyscrasia, but on the contrary, often attacks children in perfect health.

5. It is much more easily curable on the general surface than on the scalp, owing to the circumstances, than in the latter situation the fungus has obtained access to the follicles of the hair.

6. Being a purely local disease, ringworm does not require, *per se*, any constitutional treatment.

7. A purely local treatment, if efficiently pursued, is always and rapidly successful.

8. Epilation, and the use of one or other of the known parasiticides, are the measures of treatment required.

9. There is no real difference between ringworm on the general surface.

10. Ringworm, although not unfrequently causing minute vesicles, has no true analogy with herpes.—*Med. Times.*

